# Supplementary material for: Hawaiian Treeline Ecotones: Implications for Plant Community Conservation under Climate Change
Source: Plants (Basel). 2023 Dec 31;13(1):123. doi: 10.3390/plants13010123 (PMC10780311; doi:10.3390/plants13010123)
Supplement: Supplementary file 1 [file plants-13-00123-s001.zip › plants-2723853-supplementary.pdf]

Supplementary material for “Hawaiian treeline ecotones: Implications for plant community conservation under climate change”

Alison Ainsworth[1,2,*] and Donald R. Drake[1]

[1] School of Life Sciences, University of Hawai'i at M¯anoa, Honolulu, HI 96822, USA; dondrake@hawaii.edu.
[2] National Park Service, Pacific Island Inventory and Monitoring Network, Hawaii National Park, HI 96718, USA.
* Correspondence: alison_ainsworth@nps.gov; current address: National Park Service, Pacific West Region, School of Environmental and Forest Sciences, University of Washington, Seattle, WA, 98195, USA.

S1. Vegetation data conversion. The majority of plots (75%) used in analysis were installed in relatively homogenous vegetated areas (> 5 % cover) for vascular plant inventories and classification of plant communities (Green et al. 2015a, Green et al. 2015b). Plant species presence and abundance were recorded as foliar absolute cover relevé estimates (Mueller-Dombois and Ellenberg 1974).

The second data set contained 57 rectangular plots (1000 m^2) installed in wet forests, subalpine shrublands, and subalpine woodlands to assess status and detect long-term trends within upland plant communities (Ainsworth et al. 2011). Plant species presence and abundance were recorded using modified Whittaker nested plots (Ainsworth et al. 2011). Species presence and tree density data were converted to absolute cover to enable comparisons with the vascular plant inventory data. For all 57 plots, if species were identified as present in the plot according to the presence / absence data, but were not encountered along the understory (< 2 m) pole intercept transect lines then that species was added to the plot with a cover value of 0.33 %.

For 17 of the 57 plots (mostly WF plots), tree individuals were present in the plots, > 2 m tall, and recorded by diameter at breast height (DBH) as opposed to absolute cover. In order to convert these tree density measures into comparable absolute cover values the following conservative conversions were used per species per plot: (1) < 5 large trees (≥ 10 cm DBH), the number of trees was used as the percent cover value, (2) 5 – 80 large trees, the number of trees was rounded down to the closest 5 % for cover value, and (3) > 80 large trees, cover was recorded as 80 %. These conversions were generated after intensive qualitative review of each plot's GIS aerial imagery, sample plot photos, plot tree height data, and by comparing values with nearby mapping plots to ensure consistency across sampling methodologies.

At the time of this analysis, adequate allometric equations to convert DBH to canopy cover values were not available. Conversions have been developed to convert DBH and/or basal diameter and height to aboveground biomass for *Metrosideros* and others, but even these caution against widespread application due to these species wide climatic distributions (Aplet and Vitousek 1994, Litton and Kauffman 2008). For the purposes of this large-scale coarse assessment, we are confident that our conservative conversion from plot tree density values to cover class estimates warrants the inclusion of these 17 plots in the full data set.

S2. Environmental condition. Site specific data for 225 sample plots used to assess vegetation patterns across Hawaiian treeline ecotones are included (Wagner et al. 1999, Palmer 2003, Wagner et al. 2012, Giambelluca et al. 2013, Giambelluca et al. 2014). Site location data consist of volcano (**Vol**), Mauna Loa (ML) or Haleakalā (H), geographic latitude (**Lat**) and longitude (**Long**), and elevation (**EL**; m). Environmental data include mean annual temperature (**MAT**; °C), minimum and maximum temperature (**T**; °C), mean annual precipitation (**MAP**; mm), Penman-Montieth modeled potential evapotranspiration (**PET**; mm), aridity index (**AI**) calculated as MAP/PET, transpiration (**TR**; mm), wet-canopy evaporation (**WCE**; mm), soil evaporation (**SE**; mm), and evapotranspiration (**ET**; mm). Sample sites range from soil age (**SA**) less than 400 years old on Hawai‘i to over one million years old on Maui (1 = <750; 2 = 750<1500; 3 = 1500<3000; 4 = 3000<5000; 5 = 5000<13000; 6 = 13000<30000; 7 = 30000<50000; 8 = >140000) including five categories of lithology (**Lith**)(1 = aa; 2 = pahoehoe; 3 = aa+pahoehoe; 4 = sand+cobbles; 5 = tephra fallout+ash). Additional site data include sample plot size (**Size**; m^2), tree life form greater than three meters in height (**Tree**) by category (0 = treeless; 1 = <10% tree cover; 2 = ≥10% tree cover), and the community type (**Com**) each plot was assigned to using vegetation-based cluster analysis (SS = Subalpine Shrubland; SW = Subalpine Woodland; WF = Wet Forest).

Site	Vol	Lat	Long	EL	MAT	Tmin	Tmax	MAP	PET	AI	TR	WCE	SE	ET	SA	Lith	Size	Tree	Com
H.004K	ML	19.2446	-155.7931	1711	12.48	8.16	18.19	846	1719	0.49	4	173	240	417	3	3	700	2	SW
H.005K	ML	19.2457	-155.7911	1739	12.39	7.83	17.93	846	1288	0.66	58	199	210	467	3	3	700	2	SS
H.006K	ML	19.2463	-155.7903	1751	12.22	7.64	17.77	855	1300	0.66	27	144	271	441	3	3	700	2	SS
H.007K	ML	19.2480	-155.7870	1810	11.89	7.28	17.47	863	1338	0.65	2	66	361	428	3	3	700	1	SS
H.008K	ML	19.2499	-155.7834	1858	11.52	6.87	17.14	882	1390	0.63	11	97	336	444	3	3	700	2	SS
H.009K	ML	19.2509	-155.7816	1878	11.52	6.71	17.01	882	1390	0.63	11	97	336	444	3	3	700	0	SS
H.010K	ML	19.2477	-155.7803	1880	11.41	6.76	17.05	894	1206	0.74	1	49	322	372	3	3	700	0	SS
H.012K	ML	19.2381	-155.7845	1763	12.26	7.68	17.80	872	1318	0.66	93	246	171	510	3	3	700	2	SW
H.013K	ML	19.2378	-155.7848	1758	12.26	7.68	17.80	872	1318	0.66	93	246	171	510	3	3	700	2	SW
H.014K	ML	19.2409	-155.7853	1777	12.00	7.40	17.57	874	1951	0.45	13	275	172	460	3	3	700	2	SW
H.015K	ML	19.2397	-155.7884	1736	12.35	7.78	17.88	864	1912	0.45	13	285	184	481	3	3	700	2	SW
H.016K	ML	19.2404	-155.7872	1748	12.35	7.78	17.88	864	1912	0.45	13	285	184	481	3	3	700	2	SS
H.017K	ML	19.2419	-155.7842	1800	11.87	7.40	17.57	884	1959	0.45	13	265	182	459	3	3	700	2	SS
H.018K	ML	19.2435	-155.7813	1839	11.60	6.96	17.21	895	1979	0.45	16	275	185	476	3	3	700	0	SS
H.019K	ML	19.2468	-155.7774	1902	11.27	6.67	16.98	916	1449	0.63	18	131	314	462	3	3	700	0	SS
H.020K	ML	19.2368	-155.7858	1737	12.26	7.68	17.80	872	1318	0.66	93	246	171	510	3	3	700	2	SW
H.021K	ML	19.2385	-155.7838	1777	12.15	7.34	17.52	882	1906	0.46	11	258	207	476	3	3	700	2	SW
H.022K	ML	19.2334	-155.7824	1743	12.35	7.78	17.88	877	1325	0.66	95	251	170	516	3	3	700	2	SW
H.023K	ML	19.2338	-155.7817	1759	12.25	7.47	17.62	880	1600	0.55	2	111	329	441	3	3	700	0	SS
H.024K	ML	19.2357	-155.7785	1807	11.94	7.32	17.51	901	1526	0.59	5	135	292	431	3	3	700	0	SS
H.025K	ML	19.2383	-155.7745	1882	11.45	6.71	17.00	927	1481	0.63	5	116	317	438	3	3	700	0	SS
H.026K	ML	19.2395	-155.7722	1909	11.23	6.55	16.87	940	1879	0.50	3	154	335	492	3	3	700	0	SS
H.027K	ML	19.2423	-155.7678	1968	10.87	6.15	16.55	964	2079	0.46	5	161	347	512	3	3	700	0	SS
H.028K	ML	19.4009	-155.7454	2189	9.74	4.92	15.62	709	2292	0.31	4	122	261	386	3	3	700	0	SW
H.029K	ML	19.4042	-155.7450	2183	9.78	4.96	15.67	706	2324	0.30	4	126	259	388	3	3	700	2	SW

Site	Vol	Lat	Long	EL	MAT	Tmin	Tmax	MAP	PET	AI	TR	WCE	SE	ET	SA	Lith	Size	Tree	Com
H.030K	ML	19.3883	-155.7351	2332	9.37	4.61	15.16	738	1643	0.45	3	48	318	369	3	3	700	1	SW
H.031K	ML	19.2290	-155.6273	1860	11.68	6.99	17.22	2218	2384	0.93	29	356	251	635	1	3	700	0	SS
H.032K	ML	19.2550	-155.7828	1880	11.45	6.80	17.08	880	1335	0.66	40	150	248	438	1	3	700	0	SS
H.033K	ML	19.2567	-155.7651	2053	10.33	5.55	16.07	962	1418	0.68	0	19	420	438	1	3	700	0	SS
H.034K	ML	19.2560	-155.7764	1936	11.08	6.38	16.75	910	1272	0.72	0	23	374	397	1	3	700	0	SS
H.037K	ML	19.2221	-155.6227	1799	12.07	7.42	17.56	2308	2243	1.03	80	350	254	684	1	3	700	2	WF
H.038K	ML	19.2227	-155.6235	1808	12.04	7.39	17.54	2286	2181	1.05	54	380	203	637	1	3	700	2	SS
H.039K	ML	19.2236	-155.6247	1818	11.93	7.27	17.44	2273	2409	0.94	36	382	229	647	1	3	700	2	SS
H.040K	ML	19.2241	-155.6255	1818	11.93	7.27	17.44	2273	2409	0.94	36	382	229	647	1	3	700	2	SS
H.041K	ML	19.2269	-155.6292	1852	11.73	7.04	17.26	2225	2475	0.90	22	337	295	653	3	3	700	2	WF
H.042K	ML	19.2296	-155.6334	1880	11.47	6.73	17.01	2171	2277	0.95	26	303	281	610	3	3	700	2	WF
H.043K	ML	19.2650	-155.5888	1931	11.29	6.59	16.92	2120	2209	0.96	33	316	252	600	3	3	700	2	WF
H.044K	ML	19.2647	-155.5878	1920	11.39	6.70	17.01	2139	2191	0.98	31	326	240	597	3	3	700	2	SS
H.045K	ML	19.2596	-155.5812	1815	12.03	7.40	17.57	2240	1978	1.13	46	350	205	601	3	3	700	2	SS
H.046K	ML	19.2658	-155.5899	1939	11.14	6.42	16.78	2099	2243	0.94	33	320	246	598	3	3	700	1	SS
H.047K	ML	19.2687	-155.5939	1997	10.75	5.99	16.43	2039	1551	1.31	3	120	494	616	3	3	700	0	SS
H.048K	ML	19.2716	-155.5978	2048	10.43	5.64	16.15	1982	1570	1.26	3	109	505	617	2	3	700	0	SS
H.050K	ML	19.2369	-155.6426	1958	10.97	6.22	16.62	2061	1649	1.25	3	127	541	670	2	3	700	0	SS
H.051K	ML	19.2544	-155.6208	2008	10.74	5.86	16.31	2026	1439	1.41	0	37	590	626	1	3	700	2	SS
H.052K	ML	19.2522	-155.6178	1973	10.80	6.03	16.45	2040	1568	1.30	42	152	428	622	3	3	700	2	WF
H.053K	ML	19.2468	-155.6100	1893	11.49	6.78	17.06	2157	2219	0.97	39	161	224	424	6	5	700	2	WF
H.054K	ML	19.2450	-155.6075	1877	11.68	7.00	17.23	2188	2237	0.98	28	332	257	616	3	3	700	1	SS
H.055K	ML	19.2401	-155.6010	1803	12.19	7.56	17.69	2254	1947	1.16	112	363	192	667	3	3	700	2	SS
H.057K	ML	19.2403	-155.6011	1804	12.19	7.56	17.69	2254	1947	1.16	112	363	192	667	3	3	700	2	WF
H.058K	ML	19.2403	-155.6011	1804	12.19	7.56	17.69	2254	1947	1.16	112	363	192	667	3	3	700	2	SS
H.059K	ML	19.2997	-155.5434	1909	11.41	6.61	16.88	2073	2168	0.96	20	292	290	602	2	3	700	1	SS
H.060K	ML	19.3027	-155.5472	1956	11.11	6.39	16.70	2017	2223	0.91	21	282	284	587	3	3	700	0	SS
H.061K	ML	19.3054	-155.5511	2019	10.65	5.89	16.30	1958	1488	1.32	5	111	461	577	3	3	700	2	SS
H.062K	ML	19.3195	-155.5708	2292	9.40	4.56	15.00	1661	1713	0.97	1	64	531	596	2	3	700	0	SS
H.063K	ML	19.3102	-155.5581	2130	10.02	5.21	15.75	1855	2094	0.89	14	163	411	587	3	3	700	2	SS
H.064K	ML	19.2814	-155.6154	2193	9.71	4.84	15.42	1792	1800	1.00	1	83	570	655	2	3	700	0	SS
H.065K	ML	19.2796	-155.6154	2171	9.71	4.90	15.51	1792	1800	1.00	1	83	570	655	2	3	700	0	SS
H.066K	ML	19.2753	-155.6033	2101	10.11	5.29	15.86	1907	1691	1.13	4	105	518	626	2	3	700	0	SS
H.067K	ML	19.2776	-155.6062	2130	9.96	5.13	15.73	1869	1718	1.09	2	92	546	640	2	3	700	0	SS
H.068K	ML	19.2785	-155.6078	2144	9.79	5.08	15.69	1831	1684	1.09	2	84	551	636	2	3	700	0	SS
H.072K	ML	19.2030	-155.6429	1713	12.56	7.96	18.03	2351	2236	1.05	51	318	312	680	2	3	700	0	SS
H.073K	ML	19.2046	-155.6452	1735	12.44	7.83	17.92	2311	2275	1.02	43	382	259	683	2	3	700	2	WF
H.074K	ML	19.2062	-155.6474	1750	12.36	7.74	17.86	2290	2413	0.95	25	381	282	687	2	3	700	2	SS
H.075K	ML	19.2096	-155.6519	1790	12.07	7.42	17.59	2196	2322	0.95	16	298	375	688	2	3	700	2	SS
H.076K	ML	19.2790	-155.5615	1897	11.26	6.57	16.86	2232	2166	1.03	31	319	237	588	6	5	700	0	SS
H.077K	ML	19.2835	-155.5678	1985	10.96	6.03	16.43	2166	2188	0.99	30	296	254	581	6	5	700	0	SS
H.078K	ML	19.2860	-155.5716	2036	10.51	5.74	16.21	2059	1481	1.39	4	107	484	594	6	5	700	1	SS

Site	Vol	Lat	Long	EL	MAT	Tmin	Tmax	MAP	PET	AI	TR	WCE	SE	ET	SA	Lith	Size	Tree	Com
H.079K	ML	19.2897	-155.5769	2095	10.20	5.41	15.95	1983	1491	1.33	1	93	505	599	3	3	700	0	SS
H.092K	ML	19.2917	-155.5790	2131	9.99	5.18	15.76	1930	1834	1.05	5	116	483	604	3	3	700	2	WF
H.093K	ML	19.2945	-155.5828	2165	9.77	4.94	15.56	1864	1703	1.09	2	88	531	621	3	3	700	2	SS
H.094K	ML	19.2983	-155.5881	2231	9.60	4.80	15.35	1778	1766	1.01	1	77	552	629	3	3	700	2	SS
H.095K	ML	19.3266	-155.5336	2064	10.29	5.50	15.97	1870	1748	1.07	9	131	417	557	2	3	700	0	SS
H.096K	ML	19.3326	-155.5422	2203	9.60	4.83	15.38	1721	1631	1.06	7	89	450	546	3	3	700	2	SS
H.097K	ML	19.3342	-155.5443	2226	9.57	4.77	15.29	1701	1652	1.03	6	85	461	552	3	3	700	2	SS
H.098K	ML	19.3293	-155.5375	2125	9.93	5.10	15.65	1807	1824	0.99	17	147	363	527	3	3	700	2	SS
H.099K	ML	19.3313	-155.5401	2170	9.72	4.89	15.46	1764	2184	0.81	25	182	324	531	3	3	700	0	SS
H.100K	ML	19.3372	-155.5485	2291	9.39	4.62	15.08	1635	1686	0.97	2	66	501	569	3	3	700	2	SS
H.101K	ML	19.3407	-155.5535	2383	9.10	4.38	14.74	1541	1735	0.89	1	51	518	570	3	3	700	1	SS
H.105K	ML	19.2009	-155.6874	1795	12.07	7.41	17.61	1694	1708	0.99	1	141	548	690	3	3	700	0	SS
H.108K	ML	19.2001	-155.6860	1783	12.21	7.43	17.63	1691	1705	0.99	1	143	545	688	3	3	700	0	SS
H.135K	ML	19.1916	-155.7613	1705	12.45	7.89	17.98	970	2183	0.44	17	340	184	541	3	3	700	2	SS
H.136K	ML	19.1917	-155.7565	1734	12.30	7.73	17.84	1001	1510	0.66	1	86	365	451	3	3	700	0	SS
H.137K	ML	19.3785	-155.7221	2498	8.85	4.17	14.53	768	1508	0.51	0	10	383	392	3	3	700	0	SS
H.138K	ML	19.3775	-155.7242	2463	8.97	4.27	14.67	773	1628	0.47	1	26	377	404	3	3	700	1	SS
H.139K	ML	19.3775	-155.7304	2392	9.12	4.46	14.94	769	1649	0.47	3	37	365	405	3	3	700	2	SW
H.140K	ML	19.3780	-155.7359	2346	9.32	4.56	15.09	760	1636	0.46	0	36	360	397	3	3	700	0	SS
H.141K	ML	19.3793	-155.7454	2249	9.57	4.77	15.39	758	1661	0.46	2	63	326	391	3	3	700	2	SS
H.142K	ML	19.2129	-155.7726	1701	12.65	8.03	18.09	909	1922	0.47	17	325	173	515	3	3	700	2	SS
H.143K	ML	19.2130	-155.7679	1761	12.23	7.64	17.77	948	1999	0.44	13	278	206	497	3	3	700	2	SS
H.144K	ML	19.2130	-155.7665	1775	12.06	7.64	17.77	961	2008	0.44	12	258	225	495	3	3	700	2	SS
H.145K	ML	19.2130	-155.7631	1815	11.86	7.24	17.43	974	1782	0.44	3	150	360	513	3	3	700	0	SS
H.146K	ML	19.2131	-155.7584	1874	11.53	6.88	17.14	1002	1590	0.44	0	64	408	472	3	3	700	0	SS
H.147K	ML	19.4090	-155.7401	2231	9.66	4.85	15.52	699	1935	0.44	14	120	206	340	3	3	700	1	SW
H.148K	ML	19.4091	-155.7406	2223	9.66	4.85	15.52	699	1935	0.44	14	120	206	340	3	3	700	0	SW
H.149K	ML	19.4077	-155.7307	2346	9.33	4.58	15.13	698	1464	0.44	11	43	292	345	3	3	700	1	SW
H.150K	ML	19.4075	-155.7444	2176	9.85	5.01	15.75	699	2152	0.44	6	137	220	363	3	3	700	2	SW
H.151K	ML	19.4097	-155.7448	2174	9.85	5.02	15.76	696	2136	0.44	7	144	209	360	5	3	700	2	SW
H.152K	ML	19.3951	-155.7267	2438	8.99	4.29	14.71	724	1563	0.44	0	17	369	386	3	3	700	1	SS
H.153K	ML	19.4078	-155.7316	2334	9.33	4.58	15.13	698	1464	0.48	11	43	292	345	3	3	700	1	SW
H.154K	ML	19.4082	-155.7354	2293	9.49	4.71	15.32	699	1831	0.38	8	77	254	339	3	3	700	1	SW
H.2019	ML	19.4965	-155.3850	2062	10.30	5.56	15.95	1661	1776	0.94	2	113	539	654	3	3	400	0	SS
H.2020	ML	19.4954	-155.3851	2054	10.30	5.56	15.95	1661	1776	0.94	2	113	539	654	3	3	400	0	SS
H.2021	ML	19.4881	-155.3853	1993	10.85	6.01	16.33	1662	2312	0.72	32	268	370	669	3	3	400	0	SS
H.2022	ML	19.4876	-155.3827	1953	11.02	6.34	16.61	1688	2414	0.70	131	287	280	698	5	3	400	0	SS
H.2025	ML	19.4736	-155.3611	1675	12.76	8.14	18.11	1737	2230	0.78	7	258	444	709	3	3	400	0	SS
H.2149	ML	19.4780	-155.3744	1804	12.05	7.45	17.53	1711	2420	0.71	48	388	283	719	5	3	400	2	SS
H.2171	ML	19.2398	-155.6137	1882	11.60	6.91	17.15	2193	2471	0.89	24	339	271	634	3	3	400	2	WF
H.2172	ML	19.2390	-155.6123	1869	11.67	6.98	17.21	2205	2342	0.94	26	334	267	627	3	3	400	2	WF
H.2173	ML	19.3420	-155.5321	2204	9.65	4.76	15.27	1735	1958	0.89	10	125	419	554	2	3	400	1	SS

Site	Vol	Lat	Long	EL	MAT	Tmin	Tmax	MAP	PET	AI	TR	WCE	SE	ET	SA	Lith	Size	Tree	Com
H.2174	ML	19.3163	-155.5680	2247	9.53	4.70	15.20	1716	1896	0.91	9	97	464	570	3	3	400	2	SS
H.2175	ML	19.2613	-155.5957	1930	11.11	6.39	16.75	2087	1982	1.05	23	194	391	608	6	5	400	2	SS
H.2176	ML	19.2558	-155.5955	1907	11.52	6.64	16.95	2163	2226	0.97	30	332	242	603	2	3	400	2	WF
H.2177	ML	19.2474	-155.6054	1892	11.60	6.75	17.03	2178	2298	0.95	15	282	304	601	3	3	400	2	WF
H.2178	ML	19.2174	-155.6373	1830	11.95	7.29	17.47	2265	2504	0.90	18	328	328	673	6	5	400	2	WF
H.2195	ML	19.4677	-155.3786	1740	12.33	7.88	17.91	1657	1820	0.91	2	164	509	674	4	3	400	1	SW
H.2196	ML	19.4641	-155.3815	1739	12.26	7.66	17.73	1628	1913	0.85	11	228	411	649	2	3	400	0	SS
H.2197	ML	19.4617	-155.3828	1726	12.47	7.88	17.92	1624	2218	0.73	11	285	399	695	3	3	400	0	SS
H.2198	ML	19.4652	-155.3753	1698	12.67	8.10	18.10	1656	2353	0.70	8	286	392	686	4	3	400	0	SW
H.2252	ML	19.2320	-155.6496	1853	11.73	7.03	17.24	2215	2439	0.91	19	321	304	644	3	3	400	0	WF
H.2253	ML	19.2730	-155.5779	1943	11.10	6.38	16.75	2152	2264	0.95	28	313	262	602	6	5	400	0	SS
H.2254	ML	19.2777	-155.5725	1957	10.95	6.23	16.61	2156	2162	1.00	29	247	299	575	3	3	400	1	SS
H.2255	ML	19.2894	-155.5562	1937	11.24	6.54	16.84	2147	2067	1.04	27	290	263	580	6	5	400	2	WF
H.2281	ML	19.2468	-155.6016	1870	11.61	7.03	17.26	2189	2276	0.96	29	339	243	611	3	3	400	2	WF
H.2282	ML	19.2508	-155.5953	1873	11.69	6.94	17.19	2199	2233	0.98	29	327	255	610	2	3	400	2	WF
H.2292	ML	19.4047	-155.7429	2210	9.73	4.88	15.56	703	2296	0.31	4	116	264	384	4	3	400	1	SW
H.2293	ML	19.4082	-155.7429	2198	9.75	4.93	15.63	699	2094	0.33	6	123	226	355	5	3	400	0	SW
H.SS.1	ML	19.4896	-155.4117	2271	9.35	4.60	15.01	1221	1735	0.70	3	63	469	535	3	3	1000	0	SS
H.SS.10	ML	19.3109	-155.5457	2029	10.48	5.70	16.14	1914	1445	1.32	4	106	452	561	2	3	1000	2	SS
H.SS.12	ML	19.2427	-155.6535	2033	10.50	5.75	16.24	1947	1750	1.11	2	109	586	696	2	3	1000	0	SS
H.SS.13	ML	19.2633	-155.6355	2127	9.93	4.97	15.62	1872	1701	1.10	1	74	567	641	3	3	1000	0	SS
H.SS.14	ML	19.2447	-155.7090	2183	9.74	4.92	15.53	1422	2055	0.69	1	77	616	693	3	3	1000	0	SS
H.SS.15	ML	19.4062	-155.7423	2212	9.73	4.91	15.61	703	2296	0.31	4	116	264	384	3	3	1000	1	SW
H.SS.16	ML	19.4849	-155.4067	2193	9.58	4.79	15.29	1334	1721	0.77	3	82	476	560	3	3	1000	0	SS
H.SS.17	ML	19.5192	-155.4125	2491	8.69	4.05	14.20	1059	1892	0.56	1	34	487	521	5	3	1000	0	SS
H.SS.18	ML	19.4904	-155.3920	2104	10.14	5.22	15.69	1566	1670	0.94	1	88	545	634	3	3	1000	0	SS
H.SS.19	ML	19.3317	-155.5506	2267	9.43	4.65	15.13	1666	1628	1.02	2	67	499	567	2	3	1000	0	SS
H.SS.2	ML	19.4674	-155.4287	2206	9.55	4.75	15.24	1241	1649	0.75	0	50	503	553	2	3	1000	0	SS
H.SS.20	ML	19.2981	-155.5561	2026	10.58	5.82	16.24	2014	2172	0.93	20	232	334	586	3	3	1000	2	SS
H.SS.21	ML	19.2961	-155.5742	2149	9.82	4.99	15.60	1907	1624	1.17	4	94	497	595	3	3	1000	2	SS
H.SS.22	ML	19.3023	-155.5607	2091	10.20	5.41	15.92	1921	1631	1.18	10	125	430	565	3	3	1000	1	SS
H.SS.23	ML	19.2704	-155.6213	2112	9.97	5.14	15.75	1845	1741	1.06	2	94	564	659	2	3	1000	0	SS
H.SS.24	ML	19.2670	-155.6006	2017	10.67	5.90	16.35	2013	1537	1.31	2	112	507	621	2	3	1000	2	SS
H.SS.25	ML	19.3290	-155.5273	2029	10.58	5.81	16.21	1916	1962	0.98	22	226	297	544	2	3	1000	2	SS
H.SS.27	ML	19.2656	-155.6386	2151	9.75	4.93	15.55	1820	1853	0.98	1	72	640	713	3	3	1000	0	SS
H.SS.28	ML	19.2430	-155.6548	2040	10.50	5.71	16.22	1947	1750	1.11	2	109	586	696	2	3	1000	0	SS
H.SS.29	ML	19.2349	-155.7113	2099	10.16	5.36	15.93	1416	1931	0.73	0	74	628	702	3	3	1000	0	SS
H.SS.3	ML	19.5134	-155.4038	2373	8.97	4.29	14.55	1187	1837	0.65	2	52	481	534	5	3	1000	1	SS
H.SS.30	ML	19.3945	-155.7398	2259	9.56	4.77	15.40	724	1658	0.44	6	69	294	369	3	3	1000	2	SW
H.SS.4	ML	19.3265	-155.5521	2222	9.61	4.75	15.27	1738	1590	1.09	3	77	484	564	2	3	1000	2	SS
H.SS.5	ML	19.2756	-155.5834	2011	10.70	5.95	16.40	2076	2143	0.97	18	225	365	608	2	3	1000	2	SS
H.SS.6	ML	19.3140	-155.5745	2268	9.46	4.68	15.18	1695	1673	1.01	2	67	528	596	3	3	1000	0	SS

Site	Vol	Lat	Long	EL	MAT	Tmin	Tmax	MAP	PET	AI	TR	WCE	SE	ET	SA	Lith	Size	Tree	Com
H.SS.7	ML	19.3102	-155.5654	2188	9.73	4.90	15.48	1805	1870	0.97	15	127	413	555	3	3	1000	2	SS
H.SS.8	ML	19.3084	-155.5602	2138	9.97	5.02	15.60	1864	1669	1.12	3	100	486	588	3	3	1000	2	SS
H.SS.9	ML	19.3384	-155.5542	2361	9.19	4.45	14.84	1568	1748	0.90	1	55	519	575	3	3	1000	0	SS
H.WF.1	ML	19.2553	-155.5919	1877	11.58	6.90	17.16	2179	2179	1.00	31	334	235	600	6	5	1000	2	WF
H.WF.16	ML	19.2655	-155.5799	1877	11.65	6.80	17.08	2207	2132	1.04	30	333	234	596	2	3	1000	2	WF
H.WF.17	ML	19.2452	-155.6042	1870	11.79	6.91	17.16	2207	2225	0.99	30	346	235	611	3	3	1000	2	WF
H.WF.19	ML	19.2402	-155.6080	1860	11.82	7.15	17.35	2227	2302	0.97	32	358	235	625	3	3	1000	2	WF
H.WF.2	ML	19.2514	-155.6002	1899	11.45	6.75	17.03	2160	2248	0.96	26	309	277	611	3	3	1000	2	WF
H.WF.20	ML	19.2447	-155.6088	1878	11.55	6.85	17.10	2177	2268	0.96	22	297	305	623	3	3	1000	2	SS
H.WF.3	ML	19.2434	-155.6060	1848	11.72	7.03	17.26	2198	2150	1.02	28	322	232	581	3	3	1000	2	SS
H.WF.4	ML	19.2692	-155.5784	1904	11.40	6.72	17.02	2195	2142	1.02	31	322	237	590	2	3	1000	2	WF
H.WF.5	ML	19.2813	-155.5634	1941	11.26	6.42	16.75	2232	2166	1.03	31	319	237	588	6	5	1000	2	WF
M.0009	H	20.7315	-156.1437	2453	8.50	3.79	13.51	2874	1941	1.48	47	21	754	822	6	4	400	0	SS
M.0013	H	20.7340	-156.1534	2481	8.59	3.73	13.46	2391	2235	1.07	8	41	706	755	6	4	400	0	SS
M.0014	H	20.7292	-156.1505	2348	9.18	3.90	13.64	2031	1814	1.12	11	65	580	656	6	4	400	0	SS
M.0015	H	20.7264	-156.1478	2245	9.17	4.26	14.11	2237	1826	1.22	53	108	502	663	4	4	400	0	SS
M.0016	H	20.7276	-156.1439	2226	8.90	4.33	14.22	2773	1756	1.58	5	54	689	748	4	4	400	0	SS
M.0017	H	20.7265	-156.1433	2203	9.16	4.33	14.22	2743	1715	1.60	147	158	395	701	4	4	400	0	SS
M.0018	H	20.7254	-156.1441	2191	9.20	4.38	14.23	2743	2209	1.24	172	182	406	759	4	4	400	0	SS
M.0019	H	20.7234	-156.1449	2111	9.68	4.38	14.23	2430	2228	1.09	141	251	321	712	4	4	400	0	SS
M.0020	H	20.7231	-156.1437	2088	9.68	4.96	14.64	2791	2146	1.30	39	163	567	769	4	4	400	0	SS
M.0021	H	20.7164	-156.1598	2028	10.32	5.46	15.42	1471	1889	0.78	0	36	640	675	4	4	400	0	SS
M.0022	H	20.7120	-156.1608	2002	10.40	5.58	15.54	1547	1885	0.82	3	128	505	636	2	1	400	0	SS
M.0023	H	20.7092	-156.1598	1957	10.58	5.80	15.73	1590	1856	0.86	2	132	525	658	2	1	400	0	SS
M.0024	H	20.7082	-156.1581	1897	10.83	6.10	15.93	1664	1900	0.88	11	184	457	652	4	1	400	0	SS
M.0025	H	20.7128	-156.1572	1950	10.64	5.91	15.70	1625	1845	0.88	20	179	451	650	2	2	400	0	SS
M.0026	H	20.7172	-156.1474	1960	10.33	5.63	15.17	2135	2123	1.01	134	337	226	696	4	1	400	0	SS
M.0027	H	20.7193	-156.1642	2068	10.18	5.24	15.28	1437	1716	0.84	0	29	580	609	2	1	400	0	SS
M.0028	H	20.7180	-156.1588	2059	9.95	5.05	15.04	1472	1880	0.78	15	122	479	616	4	4	400	0	SS
M.0062	H	20.7225	-156.1507	2023	10.27	5.10	14.87	1610	1755	0.92	50	175	427	652	4	1	400	0	SS
M.0063	H	20.7249	-156.1929	2216	9.50	4.65	15.05	1315	1869	0.70	0	40	544	583	6	4	400	0	SS
M.0064	H	20.7407	-156.1884	2047	10.30	5.39	15.56	1846	1406	1.31	32	123	375	529	1	4	400	0	SS
M.0084	H	20.7045	-156.1575	1784	11.74	7.15	16.81	1858	1848	1.01	69	279	341	689	4	1	400	0	SS
M.0085	H	20.6995	-156.1615	1712	12.30	7.70	17.38	1735	1631	1.06	79	272	255	606	4	1	400	0	SS
M.0086	H	20.6984	-156.1594	1658	12.70	8.15	17.75	1856	1530	1.21	43	212	357	611	4	1	400	0	SS
M.0087	H	20.6973	-156.1551	1615	12.85	8.35	17.87	2068	1862	1.11	21	285	392	697	2	1	400	0	SS
M.0129	H	20.7058	-156.1845	2261	9.46	4.67	15.07	1288	1720	0.75	1	53	436	489	8	3	400	0	SS
M.0130	H	20.7063	-156.1930	2224	9.49	4.71	15.18	1248	1701	0.73	0	38	479	516	8	2	400	0	SS
M.0131	H	20.7034	-156.1969	2242	9.13	4.73	15.22	1254	2178	0.58	0	43	550	593	1	4	400	0	SS
M.0145	H	20.7184	-156.1342	1957	10.22	5.83	15.18	3958	2457	1.61	36	236	535	807	8	3	400	2	WF
M.0146	H	20.7174	-156.1288	1841	10.65	6.37	15.60	4363	2142	2.04	68	412	212	691	6	1	400	2	WF
M.0147	H	20.7276	-156.1278	2098	9.19	5.23	14.98	4832	2054	2.35	62	67	808	937	8	3	400	2	WF

Site	Vol	Lat	Long	EL	MAT	Tmin	Tmax	MAP	PET	AI	TR	WCE	SE	ET	SA	Lith	Size	Tree	Com
M.0148	H	20.7248	-156.1296	1976	10.11	5.72	15.33	4441	2286	1.94	55	378	253	686	8	3	400	2	WF
M.0149	H	20.7238	-156.1282	1931	10.26	5.92	15.47	4712	2217	2.13	58	371	275	704	8	3	400	2	WF
M.0150	H	20.7260	-156.1331	2078	9.26	4.66	14.55	3924	2264	1.73	51	158	585	793	6	4	400	2	WF
M.0152	H	20.6938	-156.1551	1531	13.55	9.09	18.53	2110	1673	1.26	133	372	237	742	2	1	400	0	SS
M.0157	H	20.7280	-156.0913	1665	11.41	7.61	16.51	10208	2313	4.41	56	449	277	781	7	4	400	0	SS
M.0158	H	20.7304	-156.0948	1668	11.25	7.62	16.61	9815	2178	4.51	135	535	67	736	1	4	400	0	SS
M.0159	H	20.7307	-156.0959	1678	11.50	7.62	16.61	9798	1999	4.90	133	515	74	721	8	3	400	0	WF
M.0160	H	20.7357	-156.1037	1850	10.55	6.45	15.82	8673	2275	3.81	155	470	85	710	5	4	400	2	WF
M.0161	H	20.7341	-156.0996	1726	11.35	7.38	16.51	9253	2039	4.54	160	528	37	724	5	1	400	2	WF
M.0162	H	20.7106	-156.1117	1504	12.41	8.53	17.17	6130	2035	3.01	174	561	87	822	6	1	400	2	WF
M.0163	H	20.7097	-156.1121	1511	12.41	8.61	17.24	6130	2035	3.01	174	561	87	822	6	1	400	2	WF
M.0180	H	20.6894	-156.1257	1553	13.15	8.90	18.01	3588	2765	1.30	110	599	233	942	8	3	400	2	SS
M.0181	H	20.6901	-156.1272	1589	12.47	8.15	17.33	3539	2730	1.30	115	564	233	911	8	3	400	2	WF
M.SS.11	H	20.7179	-156.1533	2005	10.25	5.44	15.25	1608	1810	0.89	19	132	513	664	2	2	1000	0	SS
M.SS.13	H	20.7236	-156.1899	2308	9.33	4.51	14.82	1327	2022	0.66	1	64	516	580	6	4	1000	0	SS
M.SS.14	H	20.7108	-156.1694	2099	9.87	4.98	15.18	1387	1914	0.72	6	110	465	581	4	1	1000	0	SS
M.SS.17	H	20.7200	-156.1466	2032	9.99	5.22	14.87	2059	2113	0.97	118	283	273	674	4	1	1000	0	SS
M.SS.19	H	20.7280	-156.1813	2450	8.81	3.98	13.94	1467	1870	0.78	0	22	536	557	5	1	1000	0	SS
M.SS.21	H	20.7253	-156.1431	2177	9.20	4.39	14.24	2743	2209	1.24	172	182	406	759	4	4	1000	0	SS
M.SS.23	H	20.6990	-156.1939	2319	9.11	4.41	14.73	1277	2020	0.63	0	42	481	523	8	3	1000	0	SS
M.SS.26	H	20.7076	-156.1351	2233	8.39	3.89	13.29	3745	3520	1.06	53	93	702	848	8	3	1000	0	SS
M.SS.30	H	20.7315	-156.1413	2436	8.49	3.82	13.55	3109	1977	1.57	49	24	776	848	6	4	1000	0	SS
M.SS.6	H	20.7000	-156.1764	2437	8.83	4.14	14.24	1377	2178	0.63	8	50	456	514	8	3	1000	0	SS
M.SS.8	H	20.7361	-156.1423	2340	8.87	4.11	14.01	3308	1975	1.68	25	28	800	852	6	4	1000	0	SS
M.WF.10	H	20.7317	-156.1136	2021	9.65	5.37	15.04	6969	2130	3.27	38	131	747	916	8	3	1000	2	WF
M.WF.11	H	20.7065	-156.1142	1527	12.43	8.50	17.17	5556	1879	2.96	161	493	149	803	6	1	1000	2	WF
M.WF.12	H	20.7062	-156.1126	1511	12.43	8.50	17.17	5556	1879	2.96	161	493	149	803	6	1	1000	2	WF
M.WF.16	H	20.7132	-156.1231	1663	11.58	7.45	16.30	4924	1966	2.50	155	458	178	791	6	1	1000	2	WF
M.WF.17	H	20.6933	-156.1111	1585	12.17	8.11	17.07	4913	2638	1.86	121	582	187	889	8	3	1000	2	WF
M.WF.24	H	20.6946	-156.1120	1627	11.89	7.83	16.78	5055	2579	1.96	130	565	180	875	8	3	1000	2	WF
M.WF.29	H	20.6921	-156.1104	1566	12.33	8.11	17.07	5143	2524	2.04	124	581	177	882	8	3	1000	2	WF
M.WF.8	H	20.7293	-156.0946	1708	11.25	7.37	16.37	9815	2178	4.51	135	535	67	736	6	4	1000	2	WF

Supplement References –

Ainsworth, A.; Berkowitz, P.; Jacobi, J.D.; Loh, R.K.; Kozar, K. Focal terrestrial plant communities monitoring protocol: Pacific Island Network. Natural Resource Report NPS/PACN/NRR—2011/410. National Park Service, Fort Collins, Colorado, USA, 2011.

Aplet, G.H.; Vitousek, P.M. An age-altitude matrix analysis of Hawaiian rain-forest succession. *J. Ecol.* **1994,** *82*, 137-147.

Giambelluca, T.W.; Chen, Q.; Frazier, A.G.; Price, J.P.; Chen, Y.-L.; Chu, P.-S.; Eischeid, J.K.; Departe, D.M. Online rainfall atlas of Hawaii. *Bulletin of American Meteorological Society* **2013**, *94*, 313-316.

Giambelluca, T.W.; Shuai, X.; Barnes, M.L.; Alliss, R.J.; Longman, R.J.; Miura, T.; Chen, Q.; Frazier, A.G.; Mudd, R.G.; Cuo, L.; Businger, A.D. Evapotranspiration of Hawaii. Final report submitted to the U.S. Army Corp of Engineers - Honolulu District, and the Commission on Water Resource Management, State of Hawaii, USA, 2014.

Green, K.; Hall, M.; Lopez, C.; Ainsworth, A.; Selvig, M.; Akamine, K.; Fugate, S.; Schulz, K.; Benitez, D.; Wasser, M.; Kudray, G. Vegetation mapping inventory project: Hawaii Volcanoes National Park. Fort Collins, CO, USA: National Park Service; 2015a; p.316. Report No. NPS/PACN/NRR-2015/966.

Green, K.; Schulz, K.; Lopez, C.; Ainsworth, A.; Selvig, M.; Akamine, K.; Meston, C.; Mallinson, J.W.; Urbanski, E.; Fugate, S.; Hall, M.; Kudray, G. Vegetation mapping inventory project: Haleakala National Park. Fort Collins, CO, USA: National Park Service; 2015b; p.297. Report No. NPS/PACN/NRR-2015/986.

Litton, C.M.; Kauffman, J.B. Allometric models for predicting aboveground biomass in two widespread plants in Hawaii. *Biotropica*, **2008**, *40*, 313-320.

Mueller-Dombois, D.; Ellenberg, H. *Aims and Methods of Vegetation Ecology*; Wiley & Sons: New York, USA, 1974.

Palmer, D.D. *Hawaii's Ferns and Fern Allies*; University of Hawaii Press: Honolulu, Hawaii, USA, 2003.

Wagner, W.L.; Herbst, D.R.; Sohmer, S.H. *Manual of the Flowering Plants of Hawaii*, 2nd ed; University of Hawaii Press and Bishop Museum Press: Honolulu, Hawaii, USA, 1999.

Wagner, W.L.; Herbst, D.R.; Kahn, N.; Flynn, T. *Hawaiian Vascular Plant Updates*: a supplement to the manual of flowering plants of Hawaii and Hawaii's ferns and fern allies. University of Hawaii Press: Honolulu, Hawaii, USA, 2012.
